# Supplementary figures and images for: Optimizing direct RT-LAMP to detect transmissible SARS-CoV-2 from primary nasopharyngeal swab samples
Source: PLoS One. 2020 Dec 31;15(12):e0244882. doi: 10.1371/journal.pone.0244882 (PMC7775089; doi:10.1371/journal.pone.0244882)

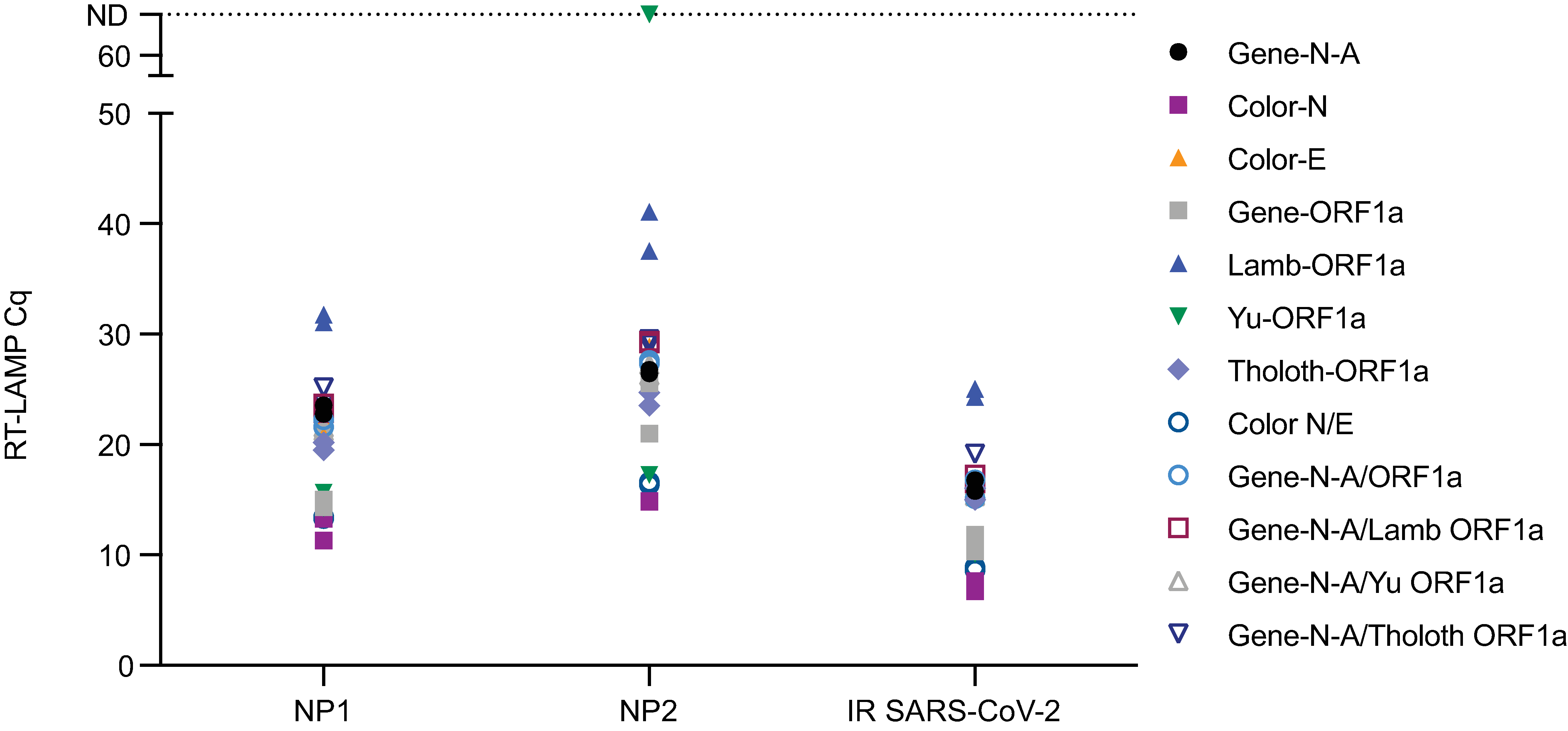

Supplement: S1 Fig — Samples that were not detectable were plotted on the ND line set at Cq 80, the hißghest cycle number in our assay. (TIF) [file pone.0244882.s001.tif]
