# Supplementary material for: Optimizing direct RT-LAMP to detect transmissible SARS-CoV-2 from primary nasopharyngeal swab samples
Source: PLoS One. 2020 Dec 31;15(12):e0244882. doi: 10.1371/journal.pone.0244882 (PMC7775089; doi:10.1371/journal.pone.0244882)
Supplement: S1 Table — (DOCX) [file pone.0244882.s002.docx]

S1 Table: Primer sequences and final 1X concentrations used in the RT-LAMP reactions.

| Primer name | | Sequence 5’->3’ | Final concentration | Pubmed ID, preprint doi, or URL for reference |
| --- | --- | --- | --- | --- |
| Gene-N-A-F3 | | TGGCTACTACCGAAGAGCT | 0.2uM | 10.1101/2020.02.26.20028373; 32300245 |
| Gene-N-A-B3 | | TGCAGCATTGTTAGCAGGAT | 0.2uM |  |
| Gene-N-A-FIP | | TCTGGCCCAGTTCCTAGGTAGTCCAGACGAATTCGTGGTGG | 1.6uM |  |
| Gene-N-A-BIP | | AGACGGCATCATATGGGTTGCACGGGTGCCAATGTGATCT | 1.6uM |  |
| Gene-N-A-LF | | GGACTGAGATCTTTCATTTTACCGT | 0.4uM |  |
| Gene-N-A-LB | | ACTGAGGGAGCCTTGAATACA | 0.4uM |  |
| As1e-F3 | | CGGTGGACAAATTGTCAC | 0.2uM | 32635743, 32900935 |
| As1e-B3 | | CTTCTCTGGATTTAACACACTT | 0.2uM |  |
| As1e-FIP | | TCAGCACACAAAGCCAAAAATTTATTTTTCTGTGCAAAGGAAATTAAGGAG | 1.6uM |  |
| As1e-BIP | | TATTGGTGGAGCTAAACTTAAAGCCTTTTCTGTACAATCCCTTTGAGTG | 1.6uM |  |
| As1e-LF | | TTACAAGCTTAAAGAATGTCTGAACACT | 0.4uM |  |
| As1e-LB | | TTGAATTTAGGTGAAACATTTGTCACG | 0.4uM |  |
| Gene-N2-F3 | | ACCAGGAACTAATCAGACAAG | 0.2uM | 32635743 |
| Gene-N2-B3 | | GACTTGATCTTTGAAATTTGGATCT | 0.2uM |  |
| Gene-N2-FIP | | TTCCGAAGAACGCTGAAGCGGAACTGATTACAAACATTGGCC | 1.6uM |  |
| Gene-N2-BIP | | CGCATTGGCATGGAAGTCACAATTTGATGGCACCTGTGTA | 1.6uM |  |
| Gene-N2-LF | | GGGGGCAAATTGTGCAATTTG | 0.4uM |  |
| Gene-N2-LB | | CTTCGGGAACGTGGTTGACC | 0.4uM |  |
| Gene-E1-F3 | | TGAGTACGAACTTATGTACTCAT | 0.2uM | 32635743 |
| Gene-E1-B3 | | TTCAGATTTTTAACACGAGAGT | 0.2uM |  |
| Gene-E1-FIP | | ACCACGAAAGCAAGAAAAAGAAGTTCGTTTCGGAAGAGACAG | 1.6uM |  |
| Gene-E1-BIP | | TTGCTAGTTACACTAGCCATCCTTAGGTTTTACAAGACTCACGT | 1.6uM |  |
| Gene-E1-LF | | CGCTATTAACTATTAACG | 0.4uM |  |
| Gene-E1-LB | | GCGCTTCGATTGTGTGCGT | 0.4uM |  |
| Color-N-F3 | | AACACAAGCTTTCGGCAG | 0.2uM | https://www.color.com/wp-content/uploads/2020/05/LAMP-Diagnostic-Assay.pdf |
| Color-N-B3 | | GAAATTTGGATCTTTGTCATCC | 0.2uM |  |
| Color-N-FIP | | TGCGGCCAATGTTTGTAATCAGCCAAGGAAATTTTGGGGAC | 1.6uM |  |
| Color-N-BIP | | CGCATTGGCATGGAAGTCACTTTGATGGCACCTGTGTAG | 1.6uM |  |
| Color-N-LF | | TTCCTTGTCTGATTAGTTC | 0.8uM |  |
| Color-N-LB | | ACCTTCGGGAACGTGGTT | 0.8uM |  |
| Color-E-F3 | | CCGACGACGACTACTAGC | 0.2uM | https://www.color.com/wp-content/uploads/2020/05/LAMP-Diagnostic-Assay.pdf |
| Color-E-B3 | | AGAGTAAACGTAAAAAGAAGGTT | 0.2uM |  |
| Color-E-FIP | | ACCTGTCTCTTCCGAAACGAATTTGTAAGCACAAGCTGATG | 1.6uM |  |
| Color-E-BIP | | CTAGCCATCCTTACTGCGCTACTCACGTTAACAATATTGCA | 1.6uM |  |
| Color-E-LF | | TCGATTGTGTGCGTACTGC | 0.8uM |  |
| Color-E-LB | | TGAGTACATAAGTTCGTAC | 0.8uM |  |
| Color-ORF1a-F3 | | TCCAGATGAGGATGAAGAAGA | 0.2uM | 32530929; https://www.color.com/wp-content/uploads/2020/05/LAMP-Diagnostic-Assay.pdf |
| Color-ORF1a-B3 | | AGTCTGAACAACTGGTGTAAG | 0.2uM |  |
| Color-ORF1a-FIP | | AGAGCAGCAGAAGTGGCACAGGTGATTGTGAAGAAGAAGAG | 1.6uM |  |
| Color-ORF1a-BIP | | TCAACCTGAAGAAGAGCAAGAACTGATTGTCCTCACTGCC | 1.6uM |  |
| Color-ORF1a-LF | | CTCATATTGAGTTGATGGCTCA | 0.8uM |  |
| Color-ORF1a-LB | | ACAAACTGTTGGTCAACAAGAC | 0.8uM |  |
| Lamb-ORF1a-F3 | TCCAGATGAGGATGAAGAAGA | | 0.2uM | 32530929 |
| Lamb-ORF1a-B3 | AGTCTGAACAACTGGTGTAAG | | 0.2uM |  |
| Lamb-ORF1a-FIP | AGAGCAGCAGAAGTGGCACAGGTGATTGTGAAGAAGAAGAG | | 1.6uM |  |
| Lamb-ORF1a-BIP | TCAACCTGAAGAAGAGCAAGAACTGATTGTCCTCACTGCC | | 1.6uM |  |
| Lamb-ORF1a-LF | CTCATATTGAGTTGATGGCTCA | | 0.4uM |  |
| Lamb-ORF1a-LB | ACAAACTGTTGGTCAACAAGAC | | 0.4uM |  |
| Yu-ORF1a-F3 | CCACTAGAGGAGCTACTGTA | | 0.2uM | 32315390 |
| Yu-ORF1a-B3 | TGACAAGCTACAACACGT | | 0.2uM |  |
| Yu-ORF1a-FIP | AGGTGAGGGTTTTCTACATCACTATATTGGAACAAGCAAATTCTATGG | | 1.6uM |  |
| Yu-ORF1a-BIP | ATGGGTTGGGATTATCCTAAATGTGTGCGAGCAAGAACAAGTG | | 1.6uM |  |
| Yu-ORF1a-LF | CAGTTTTTAACATGTTGTGCCAACC | | 0.4uM |  |
| Yu-ORF1a-LB | TAGAGCCATGCCTAACATGCT | | 0.4uM |  |
| El-Tholoth-ORF1a-F3 | TGCTTCAGTCAGCTGATG | | 0.2uM | 10.26434/chemrxiv.11860137 |
| El-Tholoth-ORF1a-B3 | TTAAATTGTCATCTTCGTCCTT | | 0.2uM |  |
| El-Tholoth-ORF1a-FIP | TCAGTACTAGTGCCTGTGCCCACAATCGTTTTTAAACGGGT | | 1.6uM |  |
| El-Tholoth-ORF1a-BIP | TCGTATACAGGGCTTTTGACATCTATCTTGGAAGCGACAACAA | | 1.6uM |  |
| El-Tholoth-ORF1a-LF | CTGCACTTACACCGCAA | | 0.8uM |  |
| El-Tholoth-ORF1a-LB | GTAGCTGGTTTTGCTAAATTCC | | 0.8uM |  |
| Gene-ORF1a-A-F3 | CTGCACCTCATGGTCATGTT | | 0.2uM | 10.1101/2020.02.26.20028373 |
| Gene-ORF1a-A-B3 | AGCTCGTCGCCTAAGTCAA | | 0.2uM |  |
| Gene-ORF1a-A-FIP | GAGGGACAAGGACACCAAGTGTATGGTTGAGCTGGTAGCAGA | | 1.6uM |  |
| Gene-ORF1a-A-BIP | CCAGTGGCTTACCGCAAGGTTTTAGATCGGCGCCGTAAC | | 1.6uM |  |
| Gene-ORF1a-A-LF | CCGTACTGAATGCCTTCGAGT | | 0.4uM |  |
| Gene-ORF1a-A-LB | TTCGTAAGAACGGTAATAAAGGAGC | | 0.4uM |  |
