# Supplementary material for: Optimizing direct RT-LAMP to detect transmissible SARS-CoV-2 from primary nasopharyngeal swab samples
Source: PLoS One. 2020 Dec 31;15(12):e0244882. doi: 10.1371/journal.pone.0244882 (PMC7775089; doi:10.1371/journal.pone.0244882)
Supplement: S2 Table — (DOCX) [file pone.0244882.s003.docx]

S2 Table: Nucleotide sequence of the RNA transcript provided by Dr. Nathan Grubaugh.

| Reference Strain: | SARS-CoV-2_USA_WA1 |
| --- | --- |
| Gene: | Nucleocapsid |
| Genome positions: | 28,068-29,430; 1363 nucleotides |
| Sequence | |
| GAAUUGUGCGUGGAUGAGGCUGGUUCUAAAUCACCCAUUCAGUACAUCGAUAUCGGUAAUUAUACAGUUUCCUGUUUACC UUUUACAAUUAAUUGCCAGGAACCUAAAUUGGGUAGUCUUGUAGUGCGUUGUUCGUUCUAUGAAGACUUUUUAGAGUAUC AUGACGUUCGUGUUGUUUUAGAUUUCAUCUAAACGAACAAACUAAAAUGUCUGAUAAUGGACCCCAAAAUCAGCGAAAUGC ACCCCGCAUUACGUUUGGUGGACCCUCAGAUUCAACUGGCAGUAACCAGAAUGGAGAACGCAGUGGGGCGCGAUCAAAAC AACGUCGGCCCCAAGGUUUACCCAAUAAUACUGCGUCUUGGUUCACCGCUCUCACUCAACAUGGCAAGGAAGACCUUAAA UUCCCUCGAGGACAAGGCGUUCCAAUUAACACCAAUAGCAGUCCAGAUGACCAAAUUGGCUACUACCGAAGAGCUACCAGA CGAAUUCGUGGUGGUGACGGUAAAAUGAAAGAUCUCAGUCCAAGAUGGUAUUUCUACUACCUAGGAACUGGGCCAGAAGC UGGACUUCCC UAUGGUG CUAACAAA GACGGCAUCAUAUGGGUUGCAACUGAGGGAGCCUUGAAUACACCAAAAGAUCACA UUGGCACCCGCAAUCCUGCUAACAAUGCUGCAAUCGUGCUACAACUUCCUCAAGGAACAACAUUGCCAAAAGGCUUCUACG CAGAAGGGAGCAGAGGCGGCAGUCAAGCCUCUUCUCGUUCCUCAUCACGUAGUCGCAACAGUUCAAGAAAUUCAACUCCA GGCAGCAGUAGGGGAACUUCUCCUGCUAGAAUGGCUGGCAAUGGCGGUGAUGCUGCUCUUGCUUUGCUGCUGCUUGACAG AUUGAACCAGCUUGAGAGCAAAAUGUCUGGUAAAGGCCAACAACAACAAGGCCAAACUGUCACUAAGAAAUCUGCUGCUGA GGCUUCUAAGAAGCCUCGGCAAAAACGUACUGCCACUAAAGCAUACAAUGUAACACAAGCUUUCGGCAGACGUGGUCCAG AACAAACCCAAGGAAAUUUUGGGGACCAGGAACUAAUCAGACAAGGAACUGAUUACAAACAUUGGCCGCAAAUUGCACAAU UUGCCCCCAGCGCUUCAGCGUUCUUCGGAAUGUCGCGCAUUGGCAUGGAAGUCACACCUUCGGGAACGUGGUUGACCUAC ACAGGUGCCAUCAAAUUGGAUGACAAAGAUCCAAAUUUCAAAGAUCAAGUCAUUUUGCUGAAUAAGCAUAUUGACGCAUAC AAAACAUUCCCACCAACAGAGCCUAAAAAGGACAAAAAGAAGAAGGCUGAUGAAACUCAAGCCUUACCGCAGAGACA | |
