# Supplementary material for: Optimizing direct RT-LAMP to detect transmissible SARS-CoV-2 from primary nasopharyngeal swab samples
Source: PLoS One. 2020 Dec 31;15(12):e0244882. doi: 10.1371/journal.pone.0244882 (PMC7775089; doi:10.1371/journal.pone.0244882)
Supplement: S3 Table — (DOCX) [file pone.0244882.s004.docx]

S3 Table: qRT-PCR N-gene viral loads and RT-LAMP Cq values from 106 primary NP swab samples run in duplicate with 1ul of swab sample or a subset of NP swab samples run in duplicate with either 1ul of primary samples treated with Lucigen QuickExtract or 5ul of extracted vRNA. R1 is replicate 1 and R2 is replicate 2. A dash indicates not detectable in our RT-LAMP assay while ND is not detectable by the clinical laboratory providing the sample.

| Sample | vRNA copies/ml | 1ul swab R1 (Cq) | 1ul swab R2 (Cq) | Lucigen R1 (Cq) | Lucigen R2 (Cq) | 5ul RNA R1 (Cq) | 5ul RNA R2 (Cq) |
| --- | --- | --- | --- | --- | --- | --- | --- |
| 1 | 738000 | - | - | 43.33 | - | 32.85 | 31.39 |
| 2 | 281000 | - | - | - | 41.78 | 31.42 | 31.68 |
| 3 | 575000 | - | 53.74 | 44.77 | 58.37 | 29.72 | 29.76 |
| 4 | 3840000 | 43.36 | 47.53 | 34.08 | 37.66 | 27.8 | 27.5 |
| 5 | 73300 | - | - | - | - | 44.45 | - |
| 6 | 10100 | - | - | - | 44.41 | 38.96 | - |
| 7 | 652000 | 67.36 | - | 38.62 | 46.43 | 30.17 | 29.76 |
| 8 | 1130000 | - | 44.47 | 40.65 | 42.72 | 29.1 | 28.51 |
| 9 | 14100 | - | - | - | - | 34.76 | - |
| 10 | 319000 | - | - | 45.5 | 47.87 | 30.37 | 31.86 |
| 11 | 2600000 | - | - | 38.02 | 44.44 | 28.92 | 28.6 |
| 12 | 574000 | - | - | 48.37 | 41.49 | 29.45 | 29.95 |
| 13 | 1080000 | 52.88 | - | 33.49 | 35.1 | 28.88 | 28.78 |
| 14 | 6.19e+008 | - | - | - | - | - | - |
| 15 | 8030000 | 38.64 | 44.91 | 41.65 | 39.68 | 26.82 | 26.78 |
| 16 | 784000 | - | - | 44.92 | - | 30.14 | 30.16 |
| 17 | 148000 | - | - | - | - | 33.23 | 34.63 |
| 18 | 78100 | - | - | - | - | 37.96 | 33.64 |
| 19 | 23700 | - | - | - | - | - | - |
| 20 | 3.35e+010 | 24.52 | 24.78 | 19.67 | 19.21 | 17.16 | 17.09 |
| 21 | 6980000 | - | 51.09 | 35.5 | 45.77 | 28.69 | 29.35 |
| 22 | 139000 | - | - | - | - | 43.07 | 36.92 |
| 23 | 9580000 | - | 54.85 | 29.85 | 32.73 | 28.15 | 28.35 |
| 24 | 6.71e+009 | 27.42 | 27.26 | 20.75 | 20.81 | 19.36 | 19.42 |
| 25 | 4.58e+009 | 30.2 | 30.06 | 22.65 | 22.37 | 20.11 | 20.19 |
| 26 | 5.98e+008 | 35.79 | 27.98 | 23.17 | 24.49 | 22.82 | 22.95 |
| 27 | 2.35e+009 | 28.6 | 29.57 | 22.72 | 22.62 | 20.98 | 20.86 |
| 28 | 5.68e+008 | 30.15 | 28.59 | 23.81 | 23.76 | 22.88 | 22.64 |
| 29 | 37600 | - | - | - | - | - | - |
| 30 | 2.59e+007 | 33.81 | 36.36 | 27.16 | 28.21 | 26.6 | 26.6 |
| 31 | 1.14e+010 | 26.86 | 23.26 | 18.39 | 18.96 | 18.66 | 18.46 |
| 32 | 29600 | - | - | - | - | 36.91 | 39.2 |
| 33 | 584000 | - | - | - | - | 33.22 | 33.92 |
| 34 | 1.12e+009 | 29.22 | 27.97 | 22.35 | 22.36 | 21.41 | 21.36 |
| 35 | 3.71e+008 | 32.39 | 29.99 | 26 | 24.98 | 23.06 | 23.01 |
| 36 | 2.2e+007 | 47.59 | - | 28.54 | 28.66 | 27.34 | 27.4 |
| 37 | 16400 | - | - | - | - | - | - |
| 38 | 1.07e+008 | 37.2 | 38.36 | 25.61 | 27.88 | 25.2 | 25.37 |
| 39 | 1.55e+008 | 34.57 | 33.19 | 25.19 | 25.74 | 25 | 24.83 |
| 40 | 7.97e+007 | 37.82 | 33.13 | 26.72 | 26.72 | 25.83 | 25.7 |
| 41 | 9870000 | 37.4 | 35.99 | 30.93 | 32.78 | 29.3 | 28.79 |
| 42 | 1.34e+008 | 30.88 | 40.14 | 25.54 | 25.01 | 24.32 | 24.17 |
| 43 | 4.79e+007 | 39.89 | 37.93 | 25.78 | 27.83 | 26.05 | 26.05 |
| 44 | 416000 | - | - | - | - | 26.05 | 42.35 |
| 45 | 473000 | - | - |  |  |  |  |
| 46 | 168000 | - | - |  |  |  |  |
| 47 | 33900 | - | - |  |  |  |  |
| 48 | 8230000 | 40.54 | 38.05 |  |  |  |  |
| 49 | 790000 | - | - |  |  |  |  |
| 50 | 139000 | - | - |  |  |  |  |
| 51 | 9870000 | 38.75 | - |  |  |  |  |
| 52 | 1380000 | - | - |  |  |  |  |
| 53 | 489000 | 36.08 | - |  |  |  |  |
| 54 | 5030000 | 37.64 | - |  |  |  |  |
| 55 | 31000 | - | - |  |  |  |  |
| 56 | 76500 | - | - |  |  |  |  |
| 57 | 4.44e+009 | 24.29 | 24.73 | 20.03 | 20.6 |  |  |
| 58 | 1.49e+009 | 26.5 | 27.33 | 21.47 | 21.69 |  |  |
| 59 | 1.84e+009 | 25.91 | 25.13 | 20.78 | 20.59 |  |  |
| 60 | 9890000 | 34.08 | 33.42 | 26.29 | 26.54 |  |  |
| 61 | 1.21e+007 | 30.34 | 35.61 | 27.92 | 21.81 |  |  |
| 62 | 2.52e+007 | 32.41 | 35.73 | 25.37 | 27.1 |  |  |
| 63 | 5010000 | 35.67 | 37.97 | 29.74 | 29.78 |  |  |
| 64 | 3.53e+007 | 31.06 | 31.26 | 23.93 | 24.79 |  |  |
| 65 | 6370000 | 33.64 | 34.33 | 28.72 | 29.75 |  |  |
| 66 | 8.03e+008 | 27.21 | 27.88 | 21.92 | 21.21 |  |  |
| 67 | 8.77e+008 | 27.65 | 27.26 | 22.08 | 22.14 |  |  |
| 68 | 2.8e+008 | 28.32 | 27.56 | 21.73 | 22.46 |  |  |
| 69 | 1.33e+008 | 29.76 | 30.17 | 24.34 | 25.2 |  |  |
| 70 | 1.03e+009 | 27.28 | 28.2 | 22.28 | 22.13 |  |  |
| 71 | 3.05e+007 | 33.77 | 36.05 | 27.93 | 27.1 |  |  |
| 72 | 4.06e+008 | 30.29 | 29.87 | 22.87 | 22.78 |  |  |
| 73 | 1.94e+009 | 27.29 | 27.07 | 22.81 | 21.52 |  |  |
| 74 | 1.22e+009 | 26.37 | 27.56 | 22.52 | 22.48 |  |  |
| 75 | 6.58e+008 | 27.98 | 27.82 | 22.31 | 22.54 |  |  |
| 76 | 1.21e+009 | 28.53 | 27.59 | 22.26 | 22.51 |  |  |
| 77 | 1.57e+009 | 27.68 | 27.78 | 22.53 | 23.74 |  |  |
| 78 | 1.34e+009 | 28.33 | 27.6 | 22.22 | 22.11 |  |  |
| 79 | 9110000 | 37.97 | 41.43 | 30.9 | 31.06 |  |  |
| 80 | 7.95e+008 | 27.94 | 28.73 | 23.06 | 23.2 |  |  |
| 81 | 6750000 | - | - | 40.59 | 37.48 |  |  |
| 82 | 1350000 | - | - | 34.49 | 41.28 |  |  |
| 83 | 9790000 | 40.64 | 44.94 | 27.6 | 30.07 |  |  |
| 84 | 6.37e+008 | 28.69 | 27.46 | 22.35 | 21.34 |  |  |
| 85 | 1.24e+007 | - | 40.41 |  |  |  |  |
| 86 | 4.59e+008 | 25.57 | 24.92 |  |  |  |  |
| 87 | 9710000 | 23.23 | 23.98 |  |  |  |  |
| 88 | 4.38e+008 | 23.63 | 25.47 |  |  |  |  |
| 89 | 1.64e+009 | 28.4 | 27.3 |  |  |  |  |
| 90 | 4650 | - | - |  |  |  |  |
| 91 | 1.52e+008 | 26.78 | 26.16 |  |  |  |  |
| 92 | 1.61e+009 | 24.71 | 25.2 |  |  |  |  |
| 93 | 7.97e+007 | 29.31 | 30.2 |  |  |  |  |
| 94 | 5.31e+007 | 41.39 | 36.57 |  |  |  |  |
| 95 | 1.42e+009 | 23.65 | 24.35 |  |  |  |  |
| 96 | 5.16e+007 | 29.88 | 33.12 |  |  |  |  |
| 97 | 6.47e+007 | 32.23 | 30.38 |  |  |  |  |
| 98 | 3.14e+009 | 23.07 | 23.14 |  |  |  |  |
| 99 | 5.89e+008 | 24.58 | 22.74 |  |  |  |  |
| 100 | 7.09e+009 | 21.3 | 24.46 |  |  |  |  |
| 101 | 3390000 | 35.59 | 74.32 |  |  |  |  |
| 102 | 2230000 | 44.09 | - |  |  |  |  |
| 103 | 1.1e+007 | 35.41 | 38.99 |  |  |  |  |
| 104 | 1.21e+007 | 36.21 | - |  |  |  |  |
| 105 | 1.66e+008 | 30.59 | 35.35 |  |  |  |  |
| 106 | 1.57e+008 | 31.51 | 34.23 |  |  |  |  |
| 107 | ND | - | - |  |  |  |  |
| 108 | ND | - | - |  |  |  |  |
| 109 | ND | - | - |  |  |  |  |
| 110 | ND | - | - |  |  |  |  |
| 111 | ND | - | - |  |  |  |  |
| 112 | ND | - | - |  |  |  |  |
| 113 | ND | - | - |  |  |  |  |
| 114 | ND | - | - |  |  |  |  |
| 115 | ND | - | - |  |  |  |  |
| 116 | ND | - | - |  |  |  |  |
| 117 | ND | - | - |  |  |  |  |
| 118 | ND | - | - |  |  |  |  |
| 119 | ND | - | - |  |  |  |  |
| 120 | ND | - | - |  |  |  |  |
| 121 | ND | - | - |  |  |  |  |
| 122 | ND | - | - |  |  |  |  |
| 123 | ND | - | - |  |  |  |  |
| 124 | ND | - | - |  |  |  |  |
| 125 | ND | - | - |  |  |  |  |
| 126 | ND | - | - |  |  |  |  |
| 127 | ND | - | - |  |  |  |  |
| 128 | ND | - | - |  |  |  |  |
| 129 | ND | - | - |  |  |  |  |
| 130 | ND | - | - |  |  |  |  |
| 131 | ND | - | - |  |  |  |  |
| 132 | ND | - | - |  |  |  |  |
| 133 | ND | - | - |  |  |  |  |
| 134 | ND | - | - |  |  |  |  |
| 135 | ND | - | - |  |  |  |  |
| 136 | ND | - | - |  |  |  |  |
| 137 | ND | - | - |  |  |  |  |
